# An X-Domain Phosphoinositide Phospholipase C (PI-PLC-like) of *Trypanosoma brucei* Has a Surface Localization and Is Essential for Proliferation

**DOI:** 10.3390/pathogens12030386

**Published:** 2023-02-28

**Authors:** Núria W. Negrão, Logan P. Crowe, Brian S. Mantilla, Rodrigo P. Baptista, Sharon King-Keller, Guozhong Huang, Roberto Docampo

**Affiliations:** 1 Center for Tropical and Emerging Global Diseases, University of Georgia, Athens, GA 30602, USA; 2 Department of Cellular Biology, University of Georgia, Athens, GA 30602, USA; 3 Department of Biosciences, Durham University, Durham DHI 3LE, UK; 4 School of Science of Technology (Biology), Georgia Gwinnett College, Lawrenceville, GA 30043, USA

**Keywords:** Phospholipase C, plasma membrane, RNA interference, *Trypanosoma brucei*

## Abstract

*Trypanosoma brucei* is the causative agent of African trypanosomiasis, a deadly disease that affects humans and cattle. There are very few drugs to treat it, and there is evidence of mounting resistance, raising the need for new drug development. Here, we report the presence of a phosphoinositide phospholipase C (TbPI-PLC-like), containing an X and a PDZ domain, that is similar to the previously characterized TbPI-PLC1. TbPI-PLC-like only possesses the X catalytic domain and does not have the EF-hand, Y, and C2 domains, having instead a PDZ domain. Recombinant TbPI-PLC-like does not hydrolyze phosphatidylinositol 4,5-bisphosphate (PIP_2_) and does not modulate TbPI-PLC1 activity in vitro. TbPI-PLC-like shows a plasma membrane and intracellular localization in permeabilized cells and a surface localization in non-permeabilized cells. Surprisingly, knockdown of *TbPI-PLC-like* expression by RNAi significantly affected proliferation of both procyclic and bloodstream trypomastigotes. This is in contrast with the lack of effect of downregulation of expression of *TbPI-PLC1*.

## 1. Introduction

Trypanosomatids are a diverse family of flagellated protozoan parasites that infect almost all vertebrate classes [1,2]. Species belonging to two genera, *Trypanosoma* and *Leishmania*, are important human pathogens causing three distinct diseases: Chagas disease, human African trypanosomiasis, and leishmaniases [2]. Despite affecting millions of people worldwide, there is a lack of effective treatments and vaccines for these diseases. Understanding the metabolic pathways of these organisms and how they differ from those in humans might lead to the identification of potential targets for drugs, diagnostics, and vaccines.

The *Trypanosoma brucei* group of parasites is responsible for causing African trypanosomiasis and has two well studied stages, the procyclic form (PCF) that replicates in the vector (*tsetse* fly) and the bloodstream form (BSF) that thrives in the blood and extracellular fluids of the mammalian host. The BSF periodically changes its surface coat of variant surface glycoprotein (VSG) by a mechanism of antigenic variation, which protects it from the host immune response [3]. VSG is attached to the external phase of the plasma membrane through a C-terminal glycosylphosphatidylinositol (GPI) anchor [4]. Release of VSG is attributed, in part, to the action of a phospholipase C [5].

Phospholipases Cs (PLCs) cleave phospholipids just before the phosphate group, and *T. brucei* has two well characterized PLCs. The first one described was named GPI-PLC [6], has a predicted molecular mass of 40 kDa, and was found to release VSG after BSF hypotonic lysis [7] or stress [8,9,10]. The enzyme cleaves the C-terminal GPI anchor of VSG, forming dimyristylglycerol and 1,2-cyclic phosphate on the inositol ring, which remains attached to the released VSG [7]. However, GPI-PLC is not essential, and its activity is not necessary for antigenic variation [11]. Different authors reported a range of subcellular localizations of GPI-PLC that were listed in [12]. More recent studies found that GPI-PLC localizes predominantly on the cytoplasmic face of the plasma membrane with concentration on the flagellar membrane and that this localization depends on acylation of cysteine residues of a CCGAC motif [13].

The second characterized PLC was termed phosphoinositide phospholipase C 1 (PI-PLC1), and it can hydrolyze phosphatidylinositol 4,5-bisphosphate (PIP_2_), generating inositol 1,4,5 trisphosphate (IP_3_) [14] and diacylglycerol, which are important signaling molecules. IP_3_ stimulates Ca^2+^ release from acidocalcisomes, which is where the IP_3_ receptor is localized [15,16]. TbPI-PLC1 has a predicted molecular mass of 80.4 kDa. In addition to conserved catalytic X and Y domains, TbPI-PLC1 has an EF-hand domain (helix-loop-helix structural domain found in calcium-binding proteins) in the N-terminal region and a C2 domain (region found in proteins that bind phospholipids in either a calcium-dependent or calcium-independent manner), as occurs with many mammalian PLCs. The domain organization of TbPI-PLC1 is more similar to that of the mammalian sperm-specific PLC-ζ protein. Sequence alignment shows an overall 27% identity and 40% similarity between the TbPI-PLC1 and mouse PLC-ζ proteins. In contrast to PLC-ζ, however, TbPI-PLC1 has an N-myristoylation consensus sequence, which is sufficient for plasma membrane localization of a fusion protein containing its first 18 amino acids. The whole protein localizes to intracellular vesicles close to the plasma membrane in PCF. TbPI-PLC1 is not essential in PCF and has a high sensitivity to Ca^2+^ with maximum activity at cytosolic Ca^2+^ levels, making it constitutively active [14].

Here, we characterized a third *T. brucei* PI-PLC, which is an ortholog of TbPI-PLC1, that was referred to for the first time as TbPI-PLC-like by Emmer et al. [17], who found that it is palmitoylated as TbPI-PLC1, or TbPI-PLC2 [14], and has a 29% identity with TbPI-PLC1. In contrast to TbPI-PLC1, TbPI-PLC-like possesses only an X catalytic domain and a PDZ domain (a protein interacting module that recognizes amino acid motifs of target proteins) instead of the Y and C2 domains and does not have an EF-hand domain. However, it has an N-terminal myristoylation domain. This protein is unable to hydrolyze PIP_2_ but is essential for growth of procyclic and bloodstream trypomastigotes.

## 2. Materials and Methods

Chemicals and reagents. Mouse antibodies against HA were from Covance (Hollywood, FL, USA). Mouse antibodies against c-Myc, Hemin, P8340 protease inhibitor, TRI reagent, *Bacillus cereus* PLC, and other analytical reagents were from Sigma-Aldrich (St. Louis, MO, USA). Goat secondary antibodies were from LI-COR Biosciences (Lincoln, NE, USA). Phusion HF DNA polymerase, P8340 protease inhibitor, other protease inhibitors, HisPur^TM^ Ni-NTA Chromatography Cartridge, and *Escherichia coli* DH5α were from Thermo Fisher Scientific (Carlsbad, CA, USA). Laemmli sample buffer was from Bio-Rad Laboratories (Hercules, CA, USA). Acrylamide mix was from National Diagnostics (Chapel Hill, NC, USA). The bicinchoninic (BCA) protein assay kit and the Silver Stain kit were from Pierce (Thermo Fisher Scientific). BenchMark Protein Ladder, Alexa-conjugated secondary antibodies, Hygromycin B, and *E. coli* BL21 Codon Plus (DE3)-RIPL were purchased from Invitrogen (Carlsbad, CA, USA). Benzonase^®^ Nuclease, anti-Histidine tag antibodies, and vector pQE-80L were from Novagen (EMD Millipore, Billerica, MA, USA). ^3^H-PIP_2_ (15–30 Ci/mmol) and ^3^H-IP_3_ (15–30 Ci/mmol) were from Perkin Elmer (Waltham, MA, USA). Fetal bovine serum (FBS) was from Atlanta Biologicals (Atlanta, GA, USA). G418 disulfate was from Teknova (Hollister, CA, USA). T4 DNA Ligase was from Promega (Madison, WI, USA). The p2T7^Ti^B/GFP vector was a gift from Dr. John Donelson (University of Iowa, Iowa City, IA, USA). Primers were ordered from IDT (Coralville, IO, USA). Restriction enzymes were from New England Biolabs (Ipswich, MA, USA).

### 2.1. Cell Cultures

Cultivation of the procyclic form (PCF) and bloodstream form (BSF) of *T. brucei* was carried out as described previously [18]. PCF Lister strain 427 were cultured at 27 °C in SDM-79 medium [19] supplemented with 10% heat-inactivated fetal bovine serum (FBS) and 7.5 μg/mL of hemin. PCF strain 29–13 (*T7RNA NEO TETR HYG*) co-expressing T7 RNA polymerase and *Tet* repressor [20] were a gift from Dr. George A. M. Cross (Rockefeller University, New York) and were cultured under the same conditions with 10% heat-inactivated tetracycline-tested FBS. G418 (15 μg/mL) and hygromycin (50 μg/mL) were added to the culture medium to maintain the integrated genes for the T7 RNA polymerase and tetracycline repressor, respectively. BSF Lister strain 427 were cultured at 37 °C, 5% CO_2_, in HMI-9 medium [21] supplemented with 10% heat-inactivated FBS. BSF single marker (BSF-SM) (*T7RNAP TETR NEO*) trypanosomes [20] were a gift from Dr. G. A. M. Cross and were grown under the same conditions with 10% heat-inactivated Tet-tested FBS and G418 (2.5 μg/mL) added to the culture medium.

### 2.2. Bioinformatic Analysis

General information available for *TbPI-PLC-like* (Tb427.06.2090 and Tb927.6.2090), *TbPI-PLC1* (Tb427tmp.02.3780 and Tb927.11.5970) and *GPI-PLC* (Tb427.02.6000 and Tb927.2.6000) and *T. brucei gambiense PI-PLC-like* (Tbg972.6.1830), *PI-PLC1* (Tbg972.11.6720) and *GPI-PLC* (Tbg972.2.6000), and human genes was obtained from TriTrypDB [22] (TriTrypDB.org) or GenBank, respectively. For the following analysis, the entire amino acid sequences deduced from the nucleotide sequences of the ORFs were used as the query. Myristoylation prediction was obtained using The MYR Predictor [23] (http://mendel.imp.ac.at/myristate/SUPLpredictor.htm accessed on 3 June 2021) and the Myristoylator [24] (web.expasy.org/myristoylator/ accessed on 3 June 3 2021). Functional analysis, classification into families, and prediction of domains was implemented using InterPro [25] (ebi.ac.uk/interpro/ accessed on 3 June 2021). We used Clustal Omega [26] (ebi.ac.uk/Tools/msa/clustalo/ accessed on 3 June 3 2021) for DNA and protein sequence alignments and to calculate identity between sequences. The I-TASSER server [27,28,29] was used to generate a structural model and predict binding partners for TbPI-PLC-like. Orthology analysis was performed using OrthoMCL DB [30] (orthomcl.org).

To reconstruct the phylogenetic PLCs found in *T. brucei* strain 927 and humans (Figure S3): TbPI-PLC1, PCL zeta (PLCζ), PLC-like and GPI-PLCs were selected from VEupathDB and Genbank databases. After the selection of their sequences, we ran Orthofinder (PMID: 31727128) against other *T. brucei* strains (*T. brucei* strain 427 and Tbg972) to obtain their respective orthologues for the phylogenetic reconstruction. These recovered amino acid sequences were then aligned using MAFFT v.7.450 [31] and submitted to Modeltest-NG [32] to select the best substitution model for the maximum likelihood reconstruction. The model selected was Jones–Taylor–Thornton (JTT) with a discrete Gamma distribution with 5 rate categories (JTT + G). The reconstruction was made using PhyML 3.3 [33] with 1000 bootstrap replicates. The tree visualization was made using Figtree (Rambaut, 2009—http://tree.bio.ed.ac.uk/software/figtree/, accessed on 3 June 2021) Comparative analysis between the *T. brucei* PLCs with their respective homologues in humans were performed by using InterproScan v.5 [34].

For the phylogenetic analysis of trypanosomatids (Figure S2), we selected all the orthologs identified by TriTrypDB and generated an alignment using MAFFT [31]. The archive with the aligned proteins was submitted to Modeltest [35] for prediction of the best model for amino acid substitution, which was the Jones–Taylor–Thornton (JTT) with Gamma distribution (+G). This model was used to generate the maximum likelihood tree using PhyML [33] with 1000 bootstrap replicates. Bootstrap values below 70% were removed for clarity as these do not have statistical support.

### 2.3. Cloning, Heterologous Expression, Purification of Recombinant TbPI-PLC-like, and Production of Antibodies

The DNA sequence of *TbPI-PLC-like* was PCR-amplified from *T. brucei* Lister strain 427 genomic DNA with specific primers carrying BamHI and HindIII restriction enzyme sites (Table S1). The purified PCR product and the prokaryotic expression vector pQE-80L (His-tag at the N-terminus) were digested overnight, gel purified, ligated, and transformed into *Escherichia coli* DH5α. After sequence verification (Genewiz, South Plainfield, NJ), positive clones were transformed into *E. coli* BL21 Codon Plus (DE3)-RIPL. Protein expression was induced with 0.5 mM isopropyl β-D-1-thiogalactopyranoside (IPTG) in Luria Bertani broth overnight at 25 °C. The protein was purified by affinity chromatography (HisPur^TM^ Ni-NTA Chromatography Cartridge), according to the manufacturer’s instructions, and identified by SDS-PAGE and Western blot analysis. The purified recombinant protein was used as an antigen to make polyclonal antibodies in mice. The antigen was injected to six female CD-1 mice (Charles River Laboratories) intraperitoneally. The primary inoculation contained 100 μg purified protein mixed in equal parts with Freund’s complete adjuvant (Sigma). Subsequent boosts, spaced in 2-week intervals, contained 50 μg purified protein mixed in equal parts with Freund’s incomplete adjuvant (Sigma). Final bleeds were collected via cardiac puncture and affinity purified by immunoadsorption to the recombinant protein immobilized on nitrocellulose strips. The adsorbed antibodies were eluted with 0.1 M glycine, pH 2.5, and neutral pH was restored immediately by adding 1 M Tris-HCL buffer, pH 8.0.

### 2.4. Enzymatic Assays

Protein concentration was determined using the BCA assay, and the recombinant protein was stored in 40% glycerol at −80 °C. The PI-PLC enzymatic activity assay was performed as described previously [36] by measuring the release of soluble IP_3_ from the hydrolysis of ^3^H-PIP_2_. Briefly, a 3:10 mixture of radioactive and cold PIP_2_ was dried under a nitrogen stream and resuspended in a reaction buffer (50 mM Hepes-NaCl, pH 7.4, 2.5 mM EGTA, 3 mM MgCl_2_, 0.2 mM DTT, 0.1% Na-deoxycholate) by sonication. Recombinant TbPI-PLC-like (20 μg) and 10 nM of Ca^2+^ were added to the reaction, and this mixture was incubated at 37 °C for 20 min. The reaction was stopped by the addition of chloroform–methanol–HCl (100:100:0.6) and 5 mM EGTA in 1 N HCl. Samples were centrifuged to separate the organic and aqueous phases. The aqueous phase was removed, and radioactivity was determined using a scintillation counter (Perkin Elmer).

### 2.5. ^3^H-IP_3_ Binding Assay

An equilibrium-competition binding assay was performed as described previously [37]. Reactions were carried out for 10 min at 4 °C in cytosol-like medium (CLM: 140 mM KCl, 20 mM NaCl, 2 mM MgCl_2_, 1 mM EGTA, and 20 mM PIPES, pH 7.0) containing radioactive IP_3_ (0.75 nM) and recombinant TbPI-PLC-like (2 μg). In some experiments, cold IP_3_ (10 µM) was added. The reactions were stopped with ice-cold CLM containing 30% poly(ethylene glycol) 8000 and γ-globulin (600 μg), followed by centrifugation (20,000× *g*, 20 min, 4 °C). The pellets were then solubilized in CLM containing 2% Triton X-100, and the radioactivity was determined using a scintillation counter.

### 2.6. Construct Design

Constructs of *TbPI-PLC-like* and *TbPI-PLC1* were subcloned from *T. brucei* Lister strain 427 genomic DNA using specific primers (Table S1). The *TbPI-PLC-like* RNAi construct was amplified using primers designed with the RNA-iT server [38], cloned into the tetracycline-inducible RNAi vector p2T7^Ti^B/GFP with dual-inducible T7 promoters (phleomycin resistance) [39], and clones were verified by sequencing (Genewiz). The one-step epitope tagging protocol reported previously [40] was used to generate cell lines with endogenous C-terminal tags. We amplified by PCR a cassette from pMOTag4H (3xHA tag; hygromycin resistance) or pMOTag23M (3xc-Myc tag; puromycin resistance) using primers that contained 80 nt homologous region of the 3’ end of the protein coding sequence and the 3’UTR of *TbPI-PLC-like* and *TbPI-PLC1*. The constructs were verified by agarose gel electrophoresis, and the PCR product was precipitated and resuspended to a concentration of 1,000 ng/μL before transfection into *T. brucei* cells.

### 2.7. Cell Transfections

*T. brucei* PCF trypanosomes in mid-log phase (~5 × 10^6^ cells/mL) were harvested by centrifugation (1000× *g*, 7 min) and washed with ice-cold Cytomix (2 mM EGTA, 3 mM MgCl_2_, 120 mM KCl, 0.5% glucose, 0.15 mM CaCl_2_, 0.1 mg/mL BSA, 10 mM K_2_HPO_4_/KH_2_PO_4_, 1 mM hypoxanthine, 25 mM Hepes, pH 7.6) and resuspended in the same buffer at a density of 2.5 × 10^7^ cells/mL. The cells were then mixed with 10 μg of NotI-linearized plasmid DNA or purified PCR products in 4 mm electroporation cuvettes and subjected to two pulses from a Bio-Rad Gene Pulser electroporator set at 1500 V, 25 μF, resting on ice 1 min in between pulses. Stable cell lines were established under drug selection with addition of phleomycin (RNAi line, 2.5 μg/mL), hygromycin (HA tag line, 50 μg/mL), or puromycin (c-Myc tag line, 1 μg/mL).

For the BSF trypanosomes, 10 μg of DNA was used per 4 × 10^7^ mid-log phase cells in 100 µL AMAXA Human T-cell Nucleofector solution (Lonza, Basel, Switzerland). Cells were electroporated in 2 mm gap cuvettes with the program X-001 of the AMAXA Nucleofector (Lonza). Following each transfection, stable transformants were selected and cloned by limiting dilution in HMI-9 medium with appropriate antibiotics (phleomycin 2.5 μg/mL, hygromycin 5 μg/mL, puromycin 0.1 μg/mL) in 24-well plates.

### 2.8. Inositol Phosphate Extraction and Analysis of Phytic Acid (IP_6_) by LC-MS

The *TbPI-PLC-like* RNAi cell lines were grown with or without 1 μg/mL tetracycline. Cell densities were determined prior to extraction and then used for data normalization. The methods for inositol phosphate extraction and analysis by LC-MS were adapted from a published protocol [41]. Analytes were detected in a Quadrupole Time-of-Flight (ToF) mass spectrometer (Micromass, Manchester, England). IPs were eluted by running a 45-min gradient of two mobile phases: buffer system A (25% MeOH: water) and system B (300 mM ammonium carbonate, pH 9.0). Phytic acid (Sigma Aldrich) was used as standard and retention time, and molecular masses from trypanosome extracts were matched. The electrospray source was set in negative-ion mode, with a spray voltage of 3000 V, ion-transfer capillary T° at 300 °C.

### 2.9. Western Blot Analyses

Parental and mutant cell lines were harvested separately, washed twice in phosphate-buffered saline (PBS), and resuspended in RIPA buffer (150 mM NaCl, 20 mM Tris-HCl, pH 7.5, 1 mM EDTA, 1% SDS, and 0.1% Triton X-100) containing a protease inhibitor cocktail (Sigma P8340) diluted 1:250, 1 mM EDTA, 1 mM phenylmethanesulfonyl fluoride (PMSF), and benzonase nuclease (25 U/mL culture). The cells were incubated on ice for 1 h and passed through an insulin syringe. The protein concentration of the lysate was determined using a BCA protein assay kit. The total cell lysate was mixed with 2 × Laemmli sample buffer at a 1:1 ratio (vol/vol) and incubated at 65 °C for 10 min. The lysates were then loaded onto a 10% SDS-PAGE. Separated proteins were transferred onto nitrocellulose membranes using a Bio-Rad transblot apparatus. Membranes were blocked with 5% (wt/vol) nonfat dried skim milk in PBS containing 0.5% Tween-20 (PBS-T) at 4 °C overnight. The blots were incubated for 1 h at 25 °C with different primary antibodies: mouse antibodies against HA (1:1000), mouse antibodies against c-Myc (1:1000), polyclonal antibodies against *TbPI-PLC-like* (1:500), rabbit antibodies against VSG221 (1:4000), and mouse antibodies against β-Tubulin (1:40,000). After five washes with PBS-T, the blots were incubated in the appropriate goat secondary antibody at a dilution of 1:15,000 and developed using an Odyssey CLx Infrared Imaging System (LI-COR) according to the manufacturer’s instructions.

### 2.10. RNA Interference

Knockdown of *TbPI-PLC-like* and *TbPI-PLC1* was induced with tetracycline in PCF and BSF cell lines carrying the RNAi cassette from p2T7^Ti^B/GFP. Transcription of the dsRNA construct was induced by the addition of 1 μg/mL tetracycline to cultures at a density of 2 × 10^6^ cells/mL (PCF) or 2 × 10^5^ cells/mL (BSF). Control cultures were grown alongside for comparison. Every other day, cell cultures were passed to fresh media to the starting density. Experiments were independently replicated on at least three different occasions. Knockdown was confirmed by reverse transcription followed by quantitative real-time polymerase chain reaction (qRT-PCR). RNA was isolated from control and induced in cultures (10^7^ cells per isolation) using TRIzol reagent, treated with DNase, and used as a template for cDNA synthesis with SuperScript III RNA Polymerase and oligo-dT (Thermo Fisher Scientific), as recommended by the manufacturer. Analysis by qRT-PCR was performed using specific primers (Table S1) and SYBR Green Supermix (Bio-Rad). The relative expression of *TbPI-PLC-like* and *TbPI-PLC1* compared to actin was calculated using the CFX Manager^TM^ Software (Bio-Rad).

### 2.11. Immunofluorescence Assays

*T. brucei* BSF and PCF were washed with Buffer A with glucose (BAG, 116 mM NaCl, 5.4 mM KCl, 0.8 mM MgSO_4_, 50 mM Hepes, pH 7.2, 5.5 mM glucose) and fixed with 2% paraformaldehyde in BAG for 1 h at 25 °C. Then they were adhered to poly-L-lysine coated coverslips and permeabilized with 0.1% Triton X-100 in PBS (pH 7.4) for 5 min. Blocking was performed overnight at 4 °C in PBS (pH 8.0) containing 100 mM NH_4_Cl, 3% BSA, 1% fish gelatin, and 5% goat serum. Cells were washed in 1% BSA in PBS (pH 8.0) and then incubated for one hour at 25°C with the following primary antibodies: mouse polyclonal anti-TbPI-PLC-like (1:25), rabbit anti-VSG221 (1:1000), rabbit anti-TcH^+^ATPase (1:50), rabbit anti-HA (1:100), and mouse anti-c-Myc (1:100). The excess primary antibody was removed with a series of washes, and the cells were incubated with the appropriate Alexa conjugated secondary antibody (1:1000) for 1 h at 25 °C. The cells were then washed and mounted to slides. DAPI (5 μg/mL) was included with the mounting medium to stain DNA. Secondary antibody controls were performed as above but in the absence of primary antibody. Differential interference contrast (DIC) and fluorescence optical images were captured with a 100 × oil immersion objective under non saturating conditions using an Olympus IX-71 inverted fluorescence microscope (Waltham, MA, USA) with a Photometrix CoolSnapHQ charge-coupled device (CCD) camera driven by DeltaVision software (Applied Precision, Issaquah, WA, USA); the images were then deconvolved for 15 cycles using Sotwarx deconvolution software.

### 2.12. Yeast Two Hybrid Assays

The Matchmaker Gold Yeast Two-Hybrid System (Takara Bio, Shiga, Japan) was used according to the manufacturer’s instructions. We used the *Saccharomyces cerevisiae* AH109 strain and standard microbial techniques and media. YPDA is 1% (wt/vol) yeast extract, 2% (wt/vol) peptone, and 2% (wt/vol) dextrose plus 100 μM adenine medium. SD medium is synthetic defined dropout medium consisting of 0.67% (wt/vol) Difco yeast nitrogen base without amino acids, 2% (wt/vol) dextrose, 2% (wt/vol) agar, 0.7% sodium phosphate dibasic, 0.3% sodium phosphate monobasic, Sunrise amino acid/nucleotide dropout mix (e.g., a complete supplement medium CSM-Leu-Trp-His-Ade dropout complete supplement mixture lacking leucine, tryptophan, histidine, and adenine), supplemented with or without 2 mM 3-amino-1,2,4-trizole (3-AT), a histidine analog and competitive inhibitor of the *His3* gene product. The full-length c-DNAs of the *TbPI-PLC-like* and *TbPI-PLC1* genes without the 5’ nucleotide sequences encoding the myristoylation consensus sequence were amplified from *T. brucei* genomic DNA by PCR using specific forward and reverse primers (Table S1) containing EcoRI and BamHI restriction sites, respectively. The PCR constructs and the YTH assay bait (pGBKT7) and prey (pGADT7) expression vectors were digested with EcoRI and BamHI overnight at 37 °C. Cloning was performed using the Gibson Assembly Kit (New England Biolabs) according to manufacturer’s protocol and positive clones were confirmed by sequencing. The recombinant YTH bait and prey plasmids were co-transformed into the yeast AH109 strain by LiOAc-mediated transformation, as described previously [42], and cultured successively on the dual, triple, and quadruple SD medium (SD-2DO medium minus Leu and Trp; SD-3DO medium minus Leu, Trp, and His; SD-4DO medium minus Leu, Trp, His, and Ade) for 3 to 4 days.

### 2.13. In Vivo Studies

To investigate the infectivity and virulence of *TbPI-PLC-like* p2T7 BSF trypanosomes we performed an in vivo study with mice. Exponentially growing cells (*TbPI-PLC-like* p2T7 +/− tetracycline for 48 h) were washed once in HMI-9 medium without selectable drugs and resuspended in the same medium. Eight-week-old Balb/c mice (6 per group) were infected with a single intraperitoneal injection of 2 × 10^4^ BSF trypanosomes in 0.2 mL of HMI-9 medium. The mice were given either normal water or water containing 200 μg/mL doxycycline in a 5% sucrose solution [18,43]. The drinking water with or without doxycycline was provided three days before infection and replaced every two days until the end of the experiment. Animals were fed ad libitum on standard chow. Parasitemia levels were monitored everyday beginning on day 3 after infection [44]. Mice were euthanized with an overdose of carbon dioxide followed by cervical dislocation when parasite density was over 1 × 10^8^ cells/mL. This study was carried out in strict accordance with the recommendations in the National Institutes of Health Guide for the Care and Use of Laboratory Animals. The animal protocol was approved by the Institutional Animal Care and Use Committee (IACUC) of the University of Georgia.

### 2.14. Statistical Analyses

All experiments were repeated at least three times with several technical replicates, as indicated in the figure legends. Results were expressed as mean values ± standard deviation (SD) or standard error of the mean (SEM). Statistical analyses were performed using GraphPad Prism software Version 8.2.0 (San Diego, CA, USA). The statistical tests used are indicated in the figure legends; the results were considered significant when *p* < 0.05 (individual *p* values are indicated in the figure legends).

## 3. Results

### 3.1. TbPI-PLC-like Sequence Analysis

**Figure 1:**
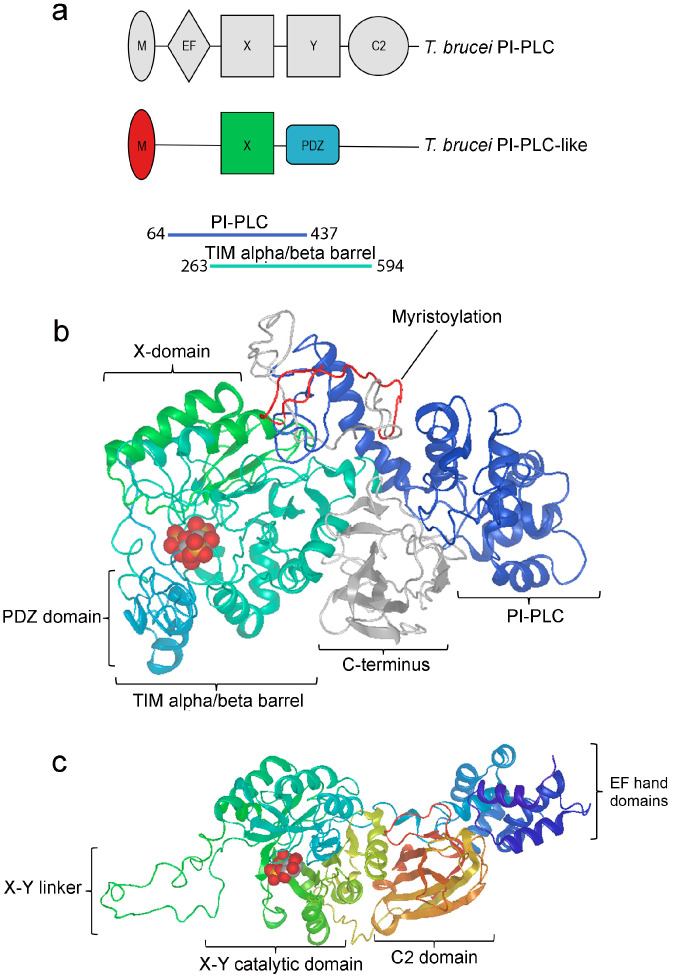
Overall structure of TbPI-PLC1, TbPI-PLC-like, and PI-PLC-ζ. (**a**) *T. brucei* PI-PLC 1 (TbPI-PLC1) has a similar domain organization to the mammalian PLC-ζ with all the canonical PLC domains, an EF-hand domain (EF), a TIM beta/alpha barrel with an X and a Y catalytic domain, and a C2 domain. In addition, it also has a myristoylation (M) consensus sequence at the N-terminus. In comparison, TbPI-PLC-like has a much simpler domain organization. It has the myristoylation consensus sequence at the N-terminus, a modified TIM beta/alpha barrel with an X domain, and a PDZ domain instead of a Y domain. TbPI-PLC-like does not have an EF-hand domain or a C2 domain. Sequence and structural similarities place TbPI-PLC-like in the PI-PLC family and TIM alpha/beta barrel phosphodiesterase superfamily. (**b**) The I-TASSER model of TbPI-PLC-like is represented in ribbon form with domain boundaries colored as in (**a**) with the myristoylation consensus sequence (membrane binding region) at the top. Shown also is IP_3_ (*red, grey,* and *gold spheres*) bound to a loop of the PDZ domain in a pocket formed by the X domain, the PDZ domain, and the rest of the TIM alpha/beta barrel sequence. The PI-PLC domain represents the sequence identified as belonging to the PI-PLC group but is not part of the other domains. (**c**) Three-dimensional protein structure model for human PLC-ζ (PDB: Q86YW0) generated by SWISS-MODEL with IP_2_ bound to the X-Y catalytic domain.

*TbPI-PLC-like* has 2130 base pairs and encodes a protein of 710 amino acids with a predicted molecular mass of 78.31 kDa. Sequence and structure similarities place *TbPI-PLC1* and *TbPI-PLC-like* in the PI-PLC and the PLC-like phosphodiesterase, TIM beta/alpha-barrel domain families, respectively. However, TbPI-PLC-like has a simpler domain architecture than other PI-PLCs (Figure 1a). TbPI-PLC-like has an N-terminus myristoylation consensus sequence (amino acids 1 to 22), lacks an EF-hand domain, has a modified TIM beta/alpha-barrel with an X catalytic domain (amino acids 322 to 395), and a PSD-95-Dlg-ZO1 (PDZ) domain (amino acids 413 to 472) instead of a Y catalytic domain, and it lacks the characteristic C2 domain on the C-terminus. The top model predicted by I-TASSER had a C-score of −0.23 (TM-score: 0.68 ± 0.12; RMSD: 8.6 ± 4.5 Å) (Figure 1b). Based on the model, IP_3_ could possibly bind to TbPI-PLC-like on a loop of the PDZ domain (C-score: 0.23), which is located on the inside of a pocket formed by the X domain, the PDZ domain, and the rest of the TIM alpha/beta barrel sequence (Figure 1b). For comparison, Figure 1c shows a model of human PLC-ζ with I(1,4)P_2_ bound to the X-Y catalytic domain.

TbPI-PLC-like belongs to the conserved (58.4% average sequence identity), kinetoplastid-specific, orthologous group OG5_151765. TriTrypDB identified 46 potential orthologs and paralogs with the same domain architecture. The sequence alignment of representative species shows high conservation in the TIM alpha/beta barrel region containing the X and PDZ domains (Figure S1). This gene is present in all kinetoplastids but not in other organisms, suggesting that a gene duplication event occurred in the common ancestor of the class. Phylogenetic analysis (Figure S2) revealed that the PI-PLC-like of *Bodo saltans* and *Paratrypanosoma confusum* are the most different from the other species. Of the trypanosomatids, the PI-PLC-like proteins of *Leishmania* species separated into one group together with *Endotrypanum monterogeii*, *Crithidia fasciculata*, and *Blechomonas ayalai. Trypanosoma* species are in a second group, with *T. cruzi* strains clustering together and with a Brazilian strain of *T. rangeli*. The African trypanosomes form their own cluster.

The orthology analysis was able to find mainly two other groups of PLCs: PI-PLCs (PLCζ in humans) and GPI-PLCs (PLCX in humans). A InterproScan analysis of all three types of PLCs found in *T. brucei* shows good correlation of domains between *T. brucei* and human PLCs (Figure S3). The GPI-PLC is most similar to the novel subgroup of PI-PLCs known as X-domain containing PI-PLC, named as PLCXD-1, -2.1, and -3, recently described in mammalian cells [45]. These enzymes contain only the X-domain of the normal X and Y catalytic domains found in mammalian PLCs and do not have any other regulatory domain.

### 3.2. Activity of Recombinant TbPI-PLC-like and Binding to IP_3_

**Figure 2:**
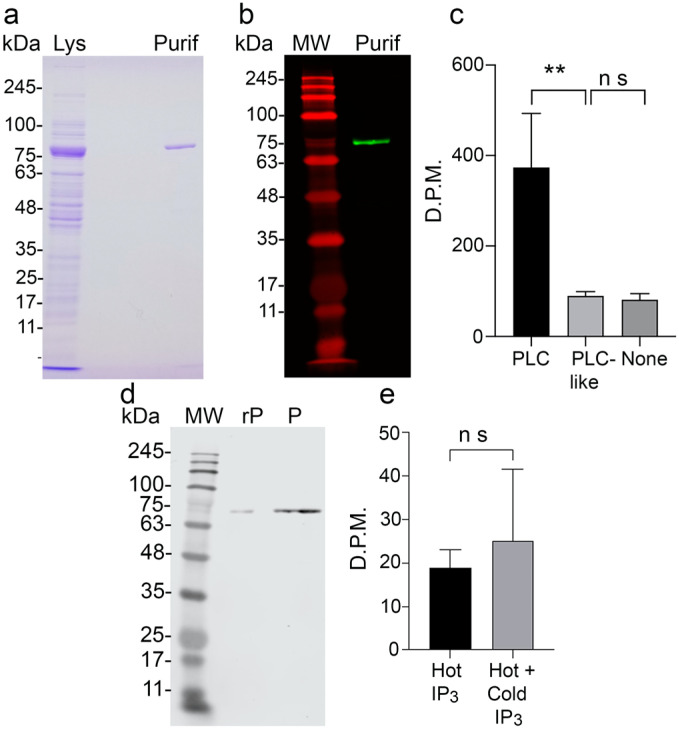
Enzymatic activity and IP_3_ binding of recombinant TbPI-PLC-like. (**a**) SDS-PAGE analysis of bacterial lysate after induction (Lys) and desalted fraction (Purif) obtained during TbPI-PLC-like affinity purification showing a band at 78.0 kDa. (**b**) Western blot analysis of the desalted fraction (Purif) collected during TbPI-PLC-like affinity purification using commercial anti-histidine tag antibody. (**c**) PIP_2_ hydrolysis by PI-PLCs in disintegrations per minute (D.P.M.). TbPI-PLC1 (PLC) was the positive control, and no enzyme was the negative control. Values are means ± s.d. from three experiments (n = 3). One-way ANOVA, ** *p* = 0.0061; ns, *p* = 0.9874. (**d**) Western blot analysis of the pellet (P) fraction of the IP_3_ binding assay compared to recombinant TbPI-PLC-like (rP) using the affinity purified mouse polyclonal antibody anti-TbPI-PLC-like. (**e**) Radioactivity of the pellet fraction of recombinant TbPI-PLC-like incubated with ^3^H-IP_3_ (*hot*), or ^3^H-IP_3_ and cold IP_3_ (*Hot + Cold*) in D.P.M. Values are means ± s.d. of three experiments (n = 3), ns: *p* = 0.6026, Students’ *t* test. MW is molecular weight.

Since TbPI-PLC-like does not have a Y catalytic domain (Figure 1a), we hypothesized that the protein would not hydrolyze PIP_2_. To characterize TbPI-PLC-like, we expressed it in *E. coli* (Figure 2a,b). TbPI-PLC-like was not able to hydrolyze PIP_2_ in vitro (Figure 2c). To investigate whether TbPI-PLC-like could bind IP_3_ we recovered recombinant TbPI-PLC-like after incubation with [^3^H]-IP_3_, or a mixture of [^3^H]-IP_3_ and cold IP_3_ (Figure 2d). However, there was no statistical difference in the amount of bound [^3^H]-IP_3_, indicating that recombinant TbPI-PLC-like does not bind to IP_3_ (Figure 2e). This result contrasts with the IP_3_ binding site predicted in the TbPI-PLC-like model (Figure 1b).

### 3.3. TbPI-PLC-like Is Not Involved in the Synthesis of Inositol Polyphosphates

IP_3_ formed by the activity of PLCs on PIP_2_ can be converted through reactions catalyzed by several kinases into inositol polyphosphates, the most abundant being inositol hexakisphosphate (IP_6_) [46]. To confirm the inability of TbPI-PLC-like to generate inositol polyphosphates in vivo, we examined the formation of IP_6_ in PCF after 48 h of downregulation of the expression of *TbPI-PLC-like* by RNAi. IP_6_ levels were measured by LC-MS (Figure S4a,b) and normalized to 3-fluoro-IP_3_. We found that there was no difference in the amount of IP_6_ (Figure S4c), indicating that TbPI-PLC-like is not involved in the inositol polyphosphate pathway.

### 3.4. Subcellular Localization of TbPI-PLC-like

**Figure 3:**
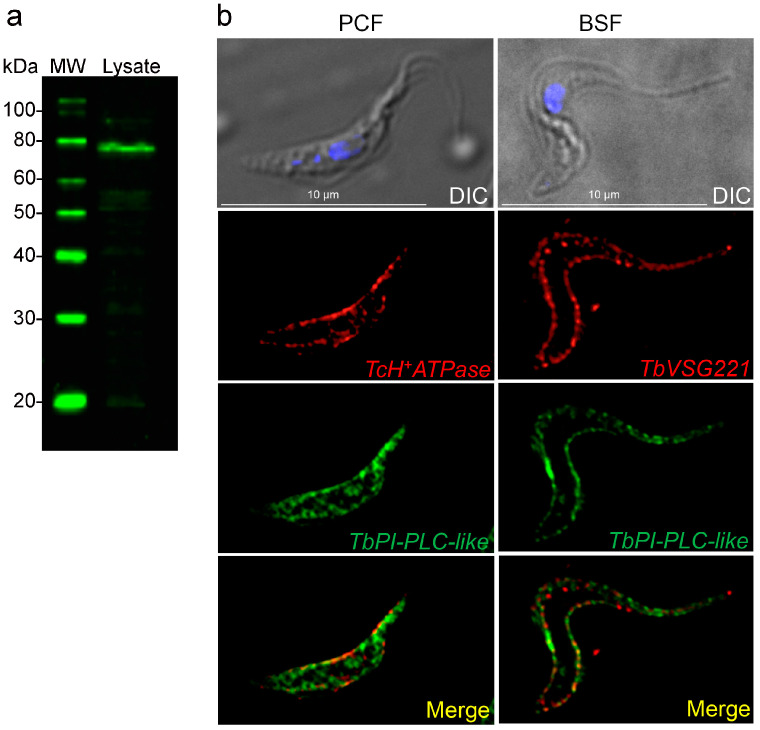
Localization of *TbPI-PLC-like*. (**a**) Western blot analysis of total cell lysate from PCF 427 WT *T. brucei* (Lysate) using affinity purified mouse polyclonal antibody against TbPI-PLC-like (1:1000), showing a band at 78.0 kDa. (**b**) Subcellular localization of TbPI-PLC-like in PCF (*left panels*) and BSF (*right panels*). In PCF, TbPI-PLC-like colocalizes with the plasma membrane marker *T. cruzi* P-type H^+^-ATPase (TcH^+^-ATPase) with a Pearson correlation coefficient of 0.8816. It also has a reticulated distribution in the cytosol. In BSF, TbPI-PLC-like colocalizes with the plasma membrane marker TbVSG-221 with a Pearson correlation coefficient of 0.6319. There is less expression in the cytosol compared to PCF. DIC, differential interference contrast microscopy.

Affinity purified antibodies were tested against total cell lysates from *T. brucei* PCF 427 WT (Figure 3a) and used to determine the subcellular localization of TbPI-PLC-like in PCF and BSF. In PCF, the protein has a reticulated distribution in the cytosol (Figure 3b). In both forms, TbPI-PLC-like has partial co-localization with membrane markers: antibodies against a plasma membrane H^+^-ATPase and against VSG 221 (Figure 3b).

### 3.5. Interaction of TbPI-PLC-like with TbPI-PLC

**Figure 4:**
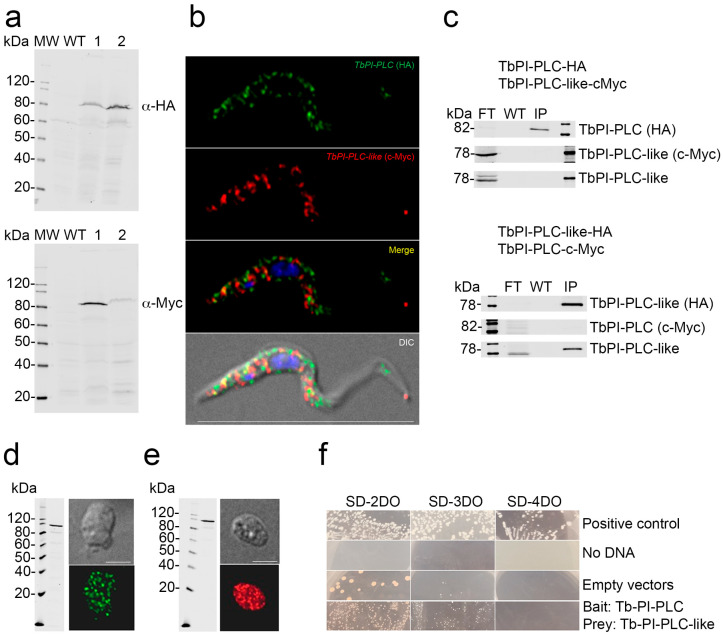
Lack of interaction between TbPI-PLC-like and TbPI-PLC1. (**a**) Western blot validation of double tagged PCF. Left lanes are molecular weight (MW) markers. Lanes 1: TbPI-PLC1 tagged with HA (82 kDa, **top panel**) or TbPI-PLC-like tagged with c-Myc (78 kDa, **bottom panel**). Lanes 2: TbPI-PLC-like tagged with HA (78 kDa, **top panel**) or TbPI-PLC1 tagged with c-Myc (82 kDa, **bottom panel**). Top panel, commercial anti-HA antibody. Bottom panel, commercial anti-c-Myc antibody. (**b**) Subcellular localization of HA tagged TbPI-PLC1 (*green*) and c-Myc tagged TbPI-PLC-like (*red*). Both tagged proteins have a punctate distribution in the cytosol and around the plasma membrane. Scale bar = 15 µm. (**c**) Western blot analysis of the pulldown with anti-HA agarose beads of TbPI-PLC1 (**top panel**) and TbPI-PLC-like (**bottom panel**) using commercial anti-HA, anti-cMyc antibodies, and anti-TbPI-PLC-like polyclonal antibody. FT: flow through, W: wash, IP: immunoprecipitate. (**d**) Western blot validation of the expression of the bait construct (*TbPI-PLC1* in pGBKT7) in the AH109 yeast cell line (**left panel**). Immunofluorescence analysis using commercial antibody against c-Myc (**right panel**) shows that the protein is expressed in the yeast cytosol. (**e**) Western blot validation of the expression of the prey construct (*TbPI-PLC-like* in pGADT) in the AH109 yeast cell line (**left panel**). Immunofluorescence analysis using commercial antibody against HA (**right panel**) shows that the protein is expressed in the cytosol. (**f**) Growth of the yeast strain AH109 expressing *TbPI-PLC1* as bait together with *TbPI-PLC-like* as prey on SD selection agar plates (SD-2DO, SD-3DO, SD-4DO). Transformations with empty vectors or without DNA were added as negative controls. Transformations with control vectors pGBKT7-53 and pGADT7-T were used as a positive control.

**Figure 5:**
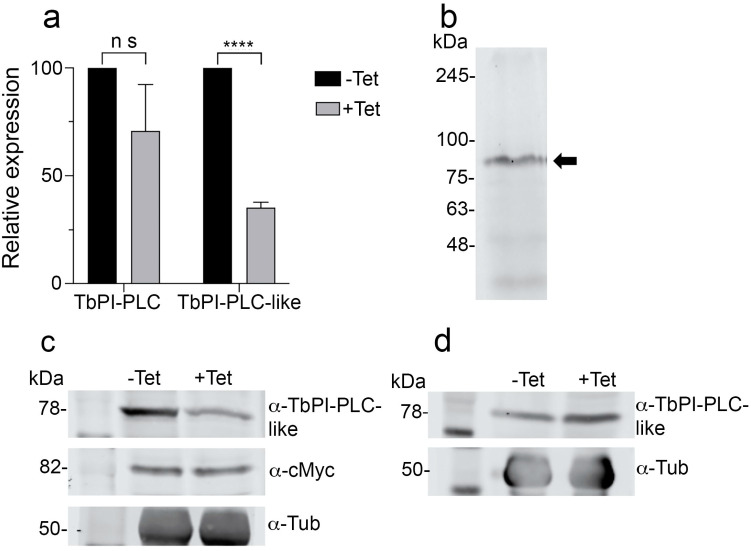
Effect of *TbPI-PLC-like* knockdown by RNAi on the expression of *TbPI-PLC1*. (**a**) qRT-PCR analysis of gene expression of *TbPI-PLC1* and *TbPI-PLC-like* relative to *actin* in *T. brucei* PCF grown in presence or absence of tetracycline for 48 h to KD the expression of *TbPI-PLC-like*. Values are means ± s.d. from three independent experiments (n = 3). **** *p* = 0.000002; ns: not significant, Students’ *t*-test. (**b**) Western blot validation of c-Myc tagged TbPI-PLC1 (82 kDa) in a *TbPI-PLC-like*-KD cell line using commercial anti-c-Myc antibody. (**c**) Western blot analysis of protein expression of *TbPI-PLC1* and *TbPI-PLC-like* in *T. brucei* PCF (cell line from (**b**)) grown in presence or absence of tetracycline for 48 h to KD the expression of *TbPI-PLC-like* using polyclonal antibody against *TbPI-PLC-like* and commercial antibody against c-Myc. Tubulin was used as a loading control. (**d**) Western blot analysis of protein expression of *TbPI-PLC1* and *TbPI-PLC-like* in *T. brucei* PCF (*TbPI-PLC*-KD cell line 29–13) grown in presence or absence of tetracycline for 48 h using polyclonal antibody against *TbPI-PLC-like*. Tubulin was used as a loading control. Results in (**b**–**d**), are representative of 3 experiments.

Previous studies in *T. brucei* reported the formation of enzyme–prozyme pairs between active enzymes and similar proteins devoid of enzymatic activity (pseudoenzymes) [47,48]. Analysis of the alignment between TbPI-PLC-like and TbPI-PLC1 by Clustal Omega revealed they are 23.15% identical, raising the possibility that they could form an enzyme–prozyme pair, as was described with other proteins in *T. brucei* [47,48]. To investigate whether the two proteins interact with each other in vivo, we used a previously generated cell line that has TbPI-PLC1 endogenously tagged with hemagglutinin (HA) [14]. The two proteins have a similar sub-cellular distribution around the plasma membrane and in the cytoplasm of PCF (Figure S5a). The overlay of the two images shows partial co-localization in vivo (Figure S5a, Merge; Pearson’s correlation coefficient of 0.6574), which does not allow us to rule out the hypothesis that the two proteins interact in vivo. Additionally, some TbPI-PLC-like co-immunoprecipitated with TbPI-PLC1 (Figure S5b). However, most of it washed out in the flow through, suggesting that the interaction might be transitory or indirect. To corroborate this weak interaction, we tested whether TbPI-PLC1 would be pulled down with TbPI-PLC-like. We used homologous recombination to produce reciprocal double-tagged cell lines with HA and c-Myc (Figure 4a). When both proteins were tagged, they did not co-localize in vivo or co-immunoprecipitate (Figure 4b,c). A yeast two hybrid screen yielded similar results; the two proteins had clear cytosolic expression in the yeast reporter cell line AH109 (Figure 4d,e), but they did not interact in vivo (Figure 4f). To test whether the two proteins have an indirect interaction, we analyzed if their expression levels were linked. We tagged TbPI-PLC1 with c-Myc in the TbPI-PLC-like-KD cell line (Figure 5b). Knocking down the expression of *TbPI-PLC-like* did not affect the expression of *TbPI-PLC1* mRNA (Figure 5a) or protein (Figure 5c). Additionally, knocking down *TbPI-PLC1* did not affect the protein expression of *TbPI-PLC-like* (Figure 5d). These results lead us to conclude that these two proteins do not interact directly or indirectly in the parasite.

### 3.6. Surface Localization of TbPI-PLC-like and TbPLC

**Figure 6:**
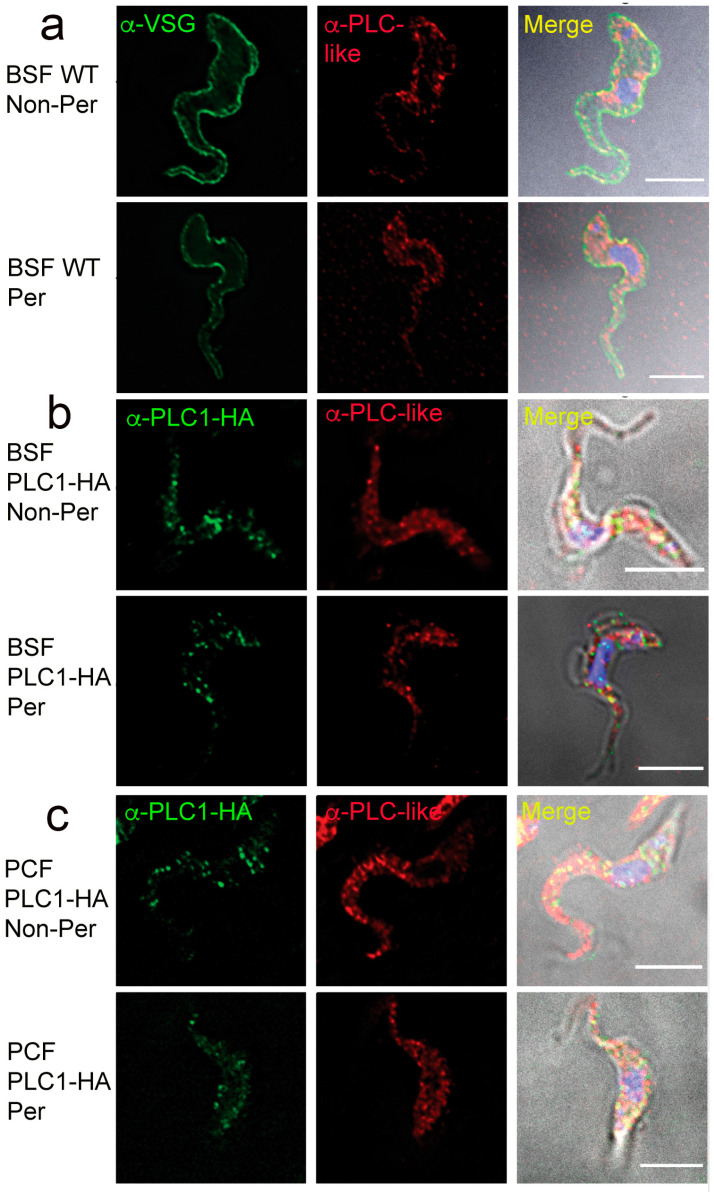
TbPI-PLC1 and TbPI-PLC-like localize to the outer surface of the plasma membrane in PCF and BSF. IFA analyses of permeabilized (bottom) and non-permeabilized (top) BSF (**a**,**b**) and PCF (**c**), expressing TbPI-PLC1-HA and TbPI-PLC-like. Partial co-localization of *TbPI-PLC-like* with a protein marker for the outer surface of the plasma membrane in BSF (VSG-221) is shown in (**a**). Representative images of parasites stained with antibodies against HA and TbPI-PLC-like show that these proteins have interactions in non-permeabilized cells in both BSF (**b**) and PCF (**c**), suggesting an outer surface localization. Scale bar = 5 μm. The images shown are a true representative of most cells but taking into consideration the morphology of the parasite. The merged images are clear because the background corresponds to the differential interference contrast (DIC) images.

The N-terminal acyl modifications of TcPI-PLC, the TbPI-PLC1 orthologue of *T. cruzi*, serve as a molecular addressing system for sending the enzyme to the outer surface of the cells [49]. As both TbPI-PLC1 and TbPI-PLC-like have a similar N-terminal acylation motifs to that of TcPI-PLC, we investigated whether they could also be expressed in the outer surface of the cells. Interestingly, we observed that both TbPI-PLC1 (C-terminally tagged with HA) and TbPI-PLC-like partially localized to the outer surface of the plasma membrane, as detected by IFA in non-permeabilized PCF and BSF (Figure 6a–c).

### 3.7. Knockdown of TbPI-PLC-like Expression

**Figure 7:**
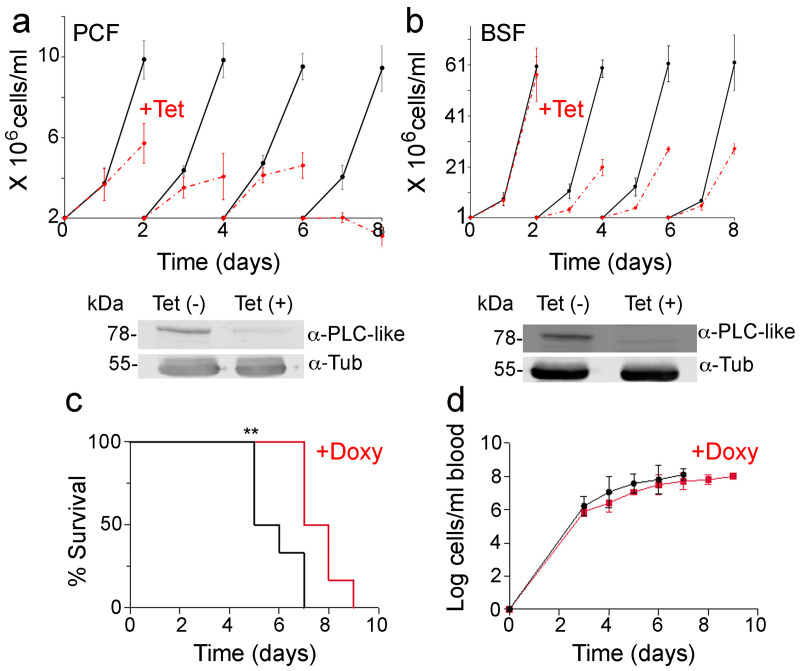
Effect of knockdown of *TbPI-PLC-like* expression on growth in vitro and in vivo. (**a**) In vitro growth of PCF 29-13 *TbPI-PLC-like*-KD cell line with (*red*) and without (*black*) 1 µg/mL tetracycline. Values are means ± s.d. of three experiments (n = 3). Day 2, ** *p* = 0.006; day 4, ** *p* = 0.002; day 6, ** *p* = 0.0006; day 8, ** *p* = 0.0003. Students’ *t*-test. Western blot shows the protein expression level of TbPI-PLC-like at 48 h of growth with or without tetracycline. Tubulin was used as a loading control. (**b**) In vitro growth of single marker BSF *TbPI-PLC-like*-KD cell line with (*red*) and without (*black*) 1 µg/mL tetracycline. Values are means ± s.d. of three experiments (n = 3). Day 4, ** *p* = 0.0001; day 6, ** *p* = 0.001; day 8, ** *p* = 0.006, Students’ *t*-test. Western blot shows the protein expression level of TbPI-PLC-like at 48 h of parasite growth with and without tetracycline. Tubulin was used as a loading control. (**c**) Two groups of six mice were infected with *TbPI-PLC-like*-KD BSF. Doxycycline (200 µg/mL) was supplied in the drinking water of one group of mice (in *red*) for the induction of RNA interference of *TbPI-PLC-like*. Percentage of survival was monitored for nine days until all mice were dead or were euthanized. ** *p* = 0.008 by the Log-rank (Mantel–Cox) test and ** *p* = 0.009 by the Gehan–Breslow–Wilcoxon test. (**d**) Parasitemia levels in the blood of infected mice were monitored for nine days until all mice were dead or were euthanized. Values are means ± s.d. (n = 6), no significant differences were determined.

Surprisingly, knockdown of *TbPI-PLC-like* by RNAi resulted in a proliferation defect in both PCF and BSF parasites (Figure 7a,b). The proliferation defect was more pronounced in PCF cells where, by day 7 of tetracycline treatment, the cells stopped growing and started dying. In BSF, the cells grew at approximately half the rate compared to the control cells. Western blot analysis revealed that TbPI-PLC-like is downregulated after two days of tetracycline treatment (Figure 7a,b). Mice infected with parasites that had *TbPI-PLC-like* knocked down survived for longer with a slightly lower parasitemia than mice infected with control parasites (Figure 7c,d). This suggests that this protein is important for virulence during infection of a mammalian host.

## 4. Discussion

In this study, we looked at a previously uncharacterized protein that was classified as a TbPI-PLC-like to see if it has a role in the inositol phosphate pathway. *TbPI-PLC-like* belongs to a highly conserved gene family in kinetoplastids, suggesting that the protein plays an important role in these organisms. The protein has general sequence and structural similarities to other PI-PLCs but has a much simpler domain organization and a modified TIM alpha/beta barrel with a PDZ domain instead of a Y domain. Interestingly, TbPI-PLC-like has no activity on PIP_2_, as TbPI-PLC1 has, but it is also able to localize to the outer surface of BSF.

PDZ are protein domains known for mediating protein–protein interactions. There are three main classes of PDZ binding domains, according to their peptide specificity, which are usually present on the C-terminus of target proteins. However, degenerate specificity and other modes of interaction were documented. For example, some PDZ domains recognize internal peptide stretches and others form homo- and hetero-dimers. PDZ peptide interactions have low micromolar affinities, and each PDZ domain can bind to more than one PDZ binding domain [50,51]. PDZ domains are also known to bind to phosphoinositides. For example, the PDZ domains of syntenin-1 and syntenin-2 bind to PIP_2_ in the plasma membrane and in the nucleus, respectively [52,53]. It is estimated that between 20% and 40% of PDZ interactions are with phospholipids [52]. The PDZ domain of TbPI-PLC-like is homologous to those found in trypsin-like serine proteases, such as DegP (HtrA), which are responsible for substrate recognition and/or binding [54]. The amino acids predicted to be involved in binding are conserved across all members of the PI-PLC-like group in kinetoplastids, indicating that this could be important for the function of the protein.

We first tested whether TbPI-PLC-like could replace TbPI-PLC1 and hydrolyze PIP_2_. We showed that the PDZ domain does not functionally replace the Y domain and that TbPI-PLC-like does not hydrolyze PIP_2_. This was expected since the Y domain of PI-PLCs is involved in the catalytic activity of the enzymes and not just in substrate binding [55]. The I-TASSER modeling server predicted that the PDZ domain of TbPI-PLC-like could bind to IP_3_, and the protein was pulled down with IP_4_ [56], suggesting that it could regulate its availability. In agreement with the lack of hydrolytic activity on PIP_2_, downregulation of the expression of *TbPI-PLC-like* did not affect the formation of IP_6_, which is the most abundant IP in the cell and is synthesized by several kinases starting from IP_3_ [46].

We then turned to our second hypothesis, that TbPI-PLC-like acted as a prozyme to TbPI-PLC1, regulating its activity in vivo, as other enzymes described in *T. brucei* [47,48]. The two proteins have a similar sub-cellular distribution; however, they do not fully co-localize. A small amount of TbPI-PLC-like consistently co-immunoprecipitated with TbPI-PLC1 despite most being lost in the flow through. The consistent finding in five independent experiments seems to indicate that the interaction between the two proteins is weak, if real. Tagging both proteins completely disrupted all interaction between the two. It is possible that the tags themselves interfered with the binding. The tags were added to the C-terminal end of both proteins and the C-terminal is sometimes involved in PDZ-mediated binding [50,51]. To more directly test whether TbPI-PLC-like regulates TbPI-PLC1 or vice versa, we looked at the expression of both proteins when either was knocked down. Knocking down *TbPI-PLC-like* did not affect the expression of *TbPI-PLC1* and knocking down *TbPI-PLC1* did not affect the expression of *TbPI-PLC-like*. This was true at the mRNA and protein levels. This strongly indicates that the two proteins are not important regulators of one another and dismisses the enzyme–prozyme hypothesis.

TbPI-PLC-like is expressed in the plasma membrane of PCF and BSF trypanosomes, and it was previously identified in a proteomics study of the flagellum of PCF parasites [57]. In PCF forms it is also prominently present in the cytosol, and another study found it in a glycosomal and mitochondrial fraction of PCF parasites but not BSF [58]. TbPI-PLC-like possesses an N-myristoylation domain, as does TbPI-PLC1, and it was shown that acylation of this domain in the *T. cruzi* orthologue TcPI-PLC addresses this protein to the extracellular phase of the plasma membrane of amastigotes [49]. This modification could explain the localization of both proteins in the outer surface of both PCF and BSF.

Surprisingly, *TbPI-PLC-like* knockdown affects the proliferation of both PCF and BSF trypanosomes. This shows that the protein has a role in both life stages, though it appears to be more important for PCF. We also showed that TbPI-PLC-like is important for virulence in a mouse model of infection. In summary, in this study we report on a PI-PLC-like protein that is highly conserved and specific to kinetoplastids. The protein has a PDZ domain and is likely to be involved in interaction with other proteins but does not regulate the activity of TbPI-PLC. We show that TbPI-PLC-like does not hydrolyze PIP_2_ and is not involved in the synthesis of inositol polyphosphates but is essential for proliferation of PCF and BSF trypanosomes.

## Data Availability

Data are contained within the article and supplementary material.

## Supplementary Materials

The following supporting information can be downloaded at: www.mdpi.com/article/10.3390/pathogens12030386/s1, Figure S1: Sequence alignment of PI-PLC proteins; Figure S2: Phylogenetic tree of PI-PLC-like proteins; Figure S3: Phylogenetic tree of trypanosomatid PI-PLCs and comparison with human PI-PLCs; Figure S4: Knockdown of *TbPI-PLC-like* expression does not affect IP_6_ production; Figure S5: Interaction between TbPI-PLC1 and TbPI-PLC-like; Table S1: Primers used in this study.
