# Supplementary material for: An X-Domain Phosphoinositide Phospholipase C (PI-PLC-like) of Trypanosoma brucei Has a Surface Localization and Is Essential for Proliferation
Source: Pathogens. 2023 Feb 28;12(3):386. doi: 10.3390/pathogens12030386 (PMC10051276; doi:10.3390/pathogens12030386)
Supplement: Supplementary file 1 [file pathogens-12-00386-s001.zip › pathogens-2219371-supplementary.pdf]

pathogens

Supplementary Information

An X-Domain Phosphoinositide Phospholipase C (PI-PLC-like) of *Trypanosoma brucei* Has a Surface Localization and Is Essential for Proliferation

Núria W. Negrão [1,2], Brian S. Mantilla [1,3], Rodrigo P. Baptista [1], Sharon King-Keller [1,4], Guozhong Huang [1] and Roberto Docampo [1,2,*]

1 Center for Tropical and Emerging Global Diseases, University of Georgia, Athens, GA 30602, USA
2 Department of Cellular Biology, University of Georgia, Athens, GA 30602, USA
3 Department of Biosciences, Durham University, DHI 3LE Durham, UK
4 Georgia Gwinnett College, Lawrenceville, GA 30043, USA
* Correspondence: rdocampo@uga.edu

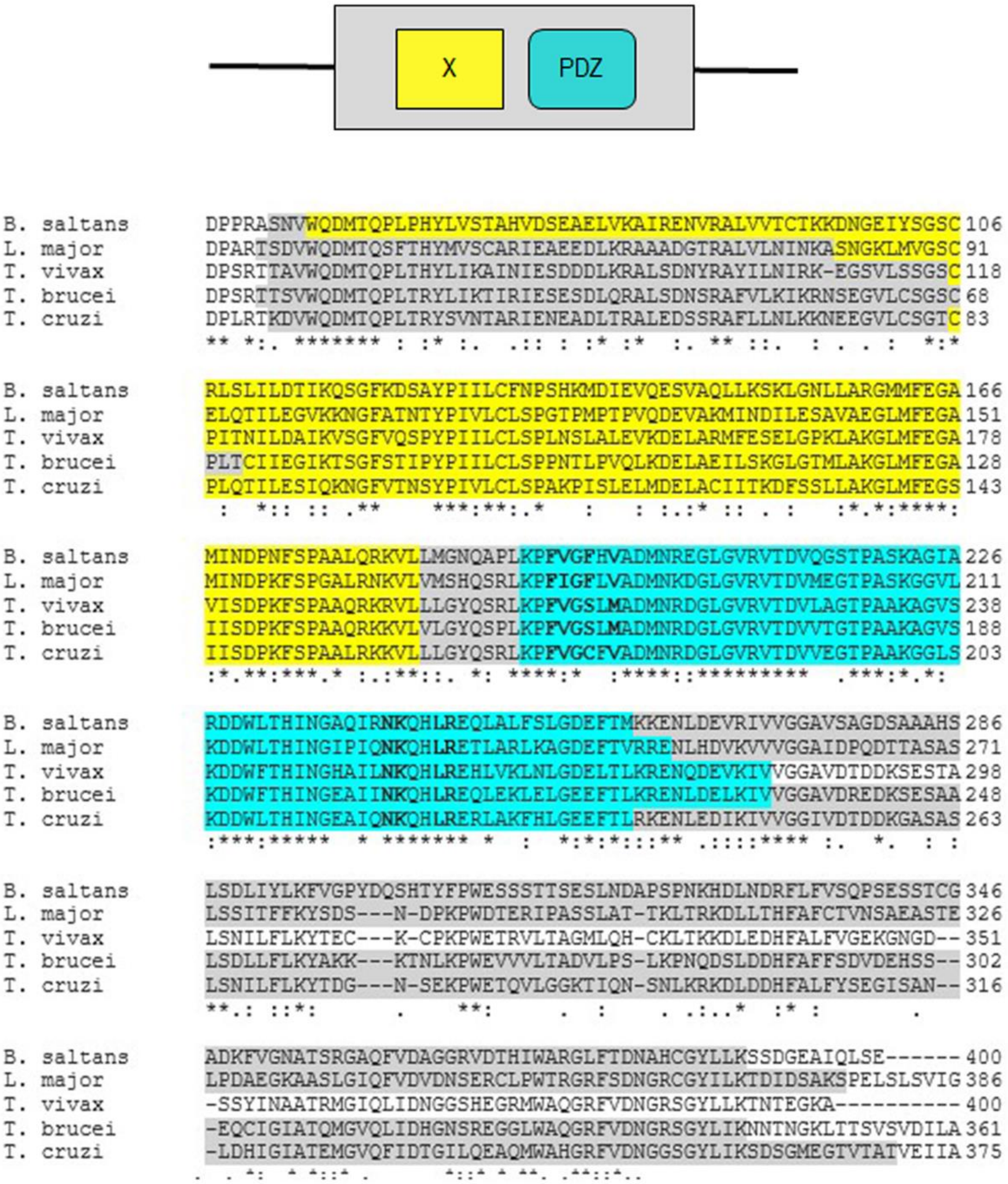

Figure S1. Sequence alignment of PI-PLC-like proteins. Alignment of the amino acid sequences of the TIM alpha/beta barrel region of PI-PLC-like proteins of representative kinetoplastid species. The amino acids corresponding to the TIM alpha/beta barrel are colored in grey, the X domain in yellow, and the PDZ domain in blue. Residues of the PDZ domain predicted to be important for protein-protein interaction are bolded.

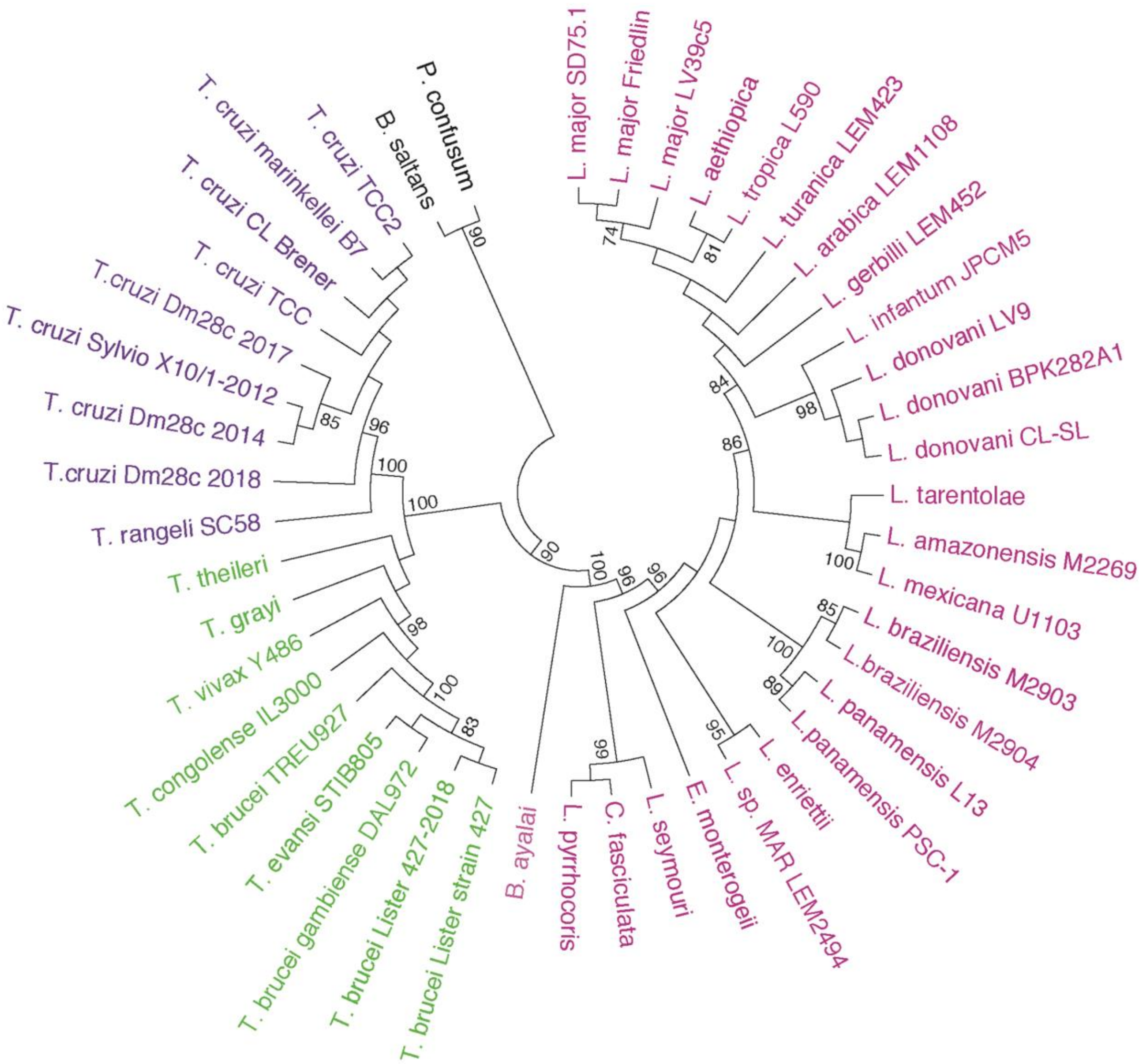

Figure S2. Phylogenetic tree of TbPI-PLC-like orthologs. Maximum likelihood tree showing how orthologs of TbPI-PLC-like cluster. The phylogeny was made using PhyML with 1000 bootstrap replicates using the JTT amino acid replacement model. *B. saltans* and *P. confusum* form an outgroup (*black*). *T. cruzi* isolates cluster together with *T. rangeli* (isolate from Brazil – purple group). The African trypanosomes form their own group (*green*). While *Leishmania* spp. cluster with other kinetoplastids (*pink*).

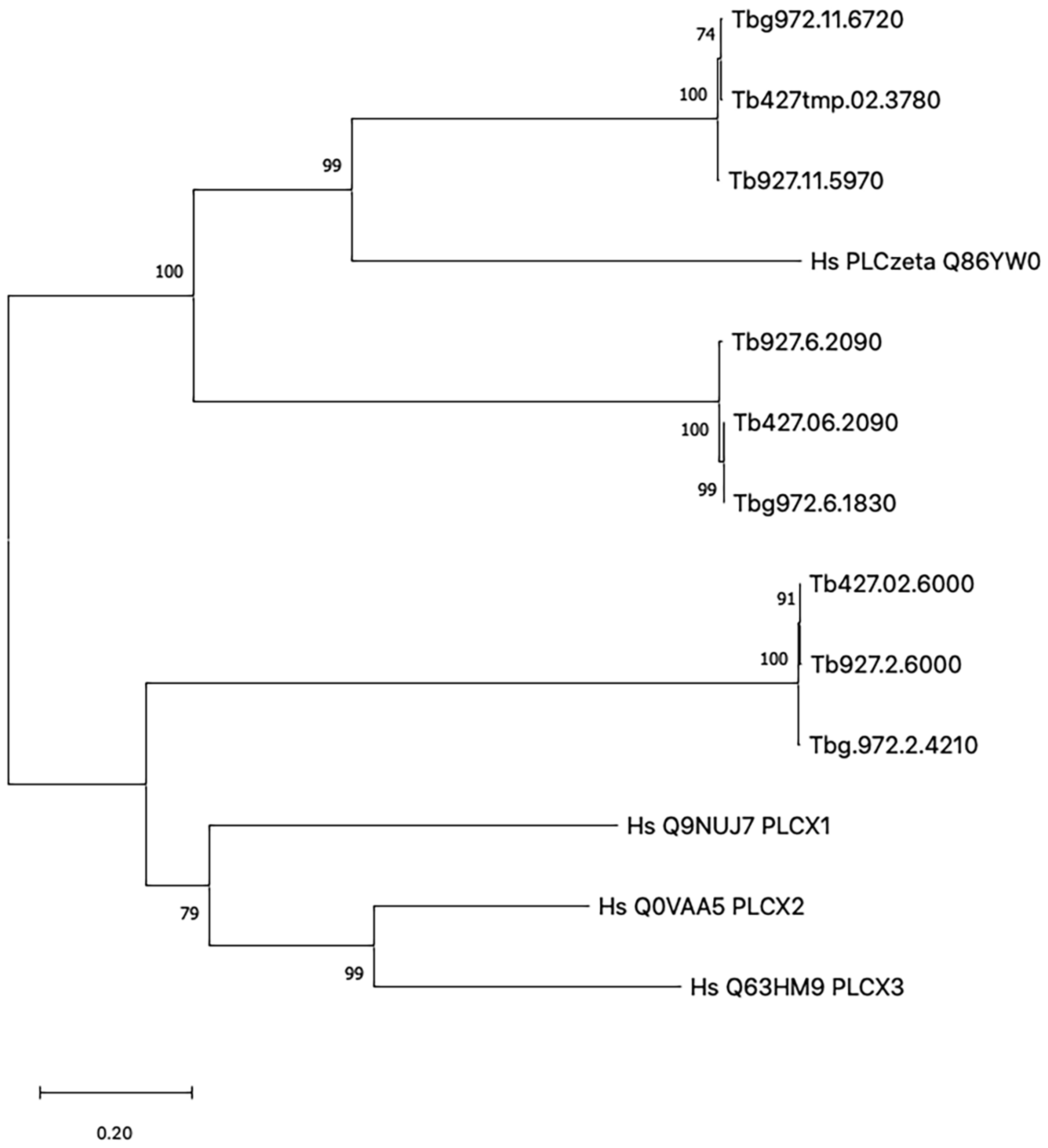

Figure S3. Phylogenetic tree of trypanosomatid PI-PLCs and comparison with PI-PLC-like, and with human PI-PLC-ζ and PLCXDs. TbPI-PLC1 (Tb427tmp.02380 and Tb927.11.5970) and *T. brucei gambiense* PLC (TbgPI-PLC; Tbg972.11.6720) are closer to *Homo sapiens* PI-PLC (HsPI-PLC-ζ, Q86YWO) than to *T. brucei (*Tb927.6.2090 and Tbg427.06.2090) and *T. brucei gambiense* (Tbg972.6.1830) PI-PLC-like. TbGPI-PLC (Tb427.02.6000 and Tb927.2.6000) and TbgGPI-PLC (Tbg972.2.6000) are closer to human PLCDX proteins 1 (Q9NUJ7), 2 (Q0VAA5), and 3 (Q63HM9).

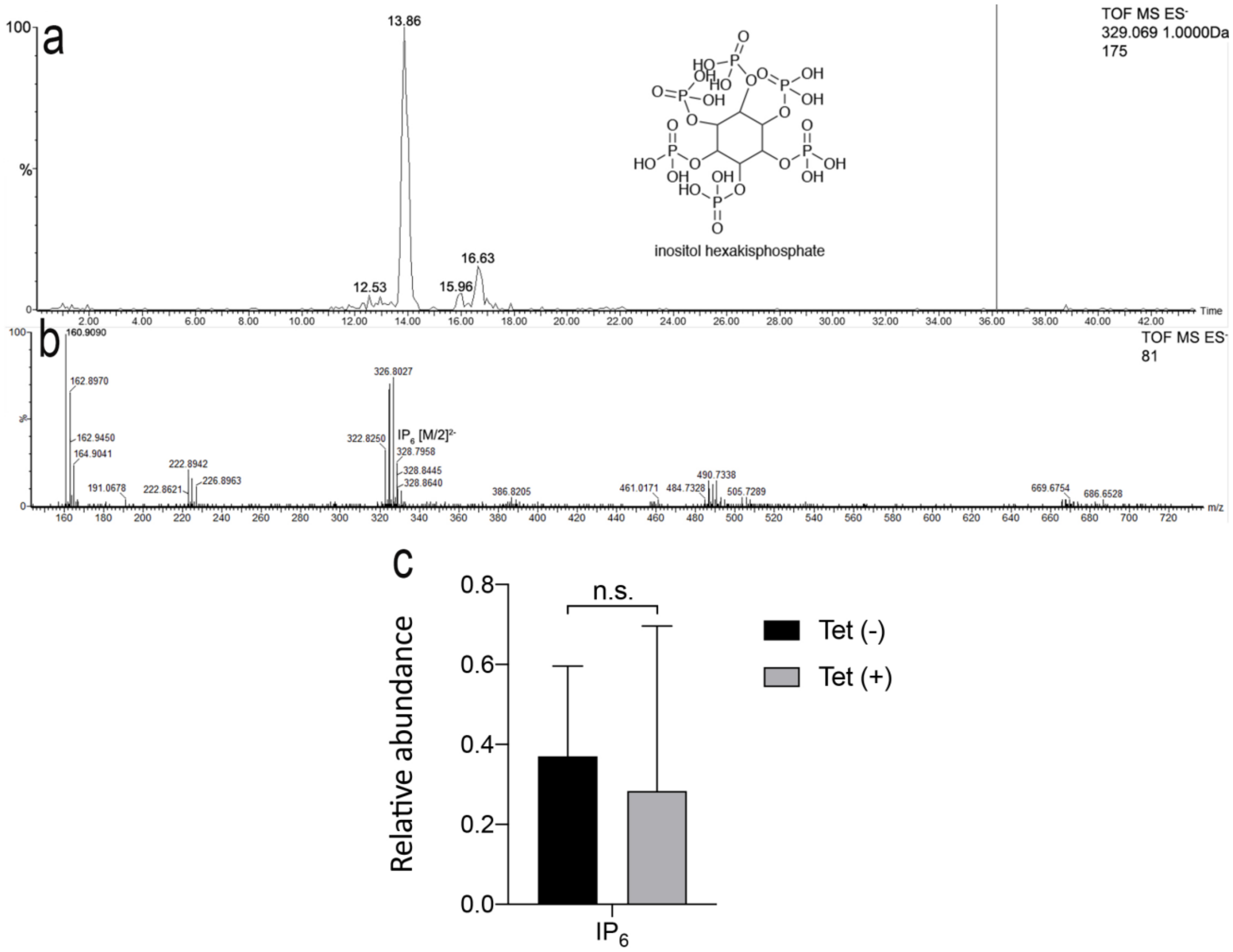

Figure S4. Knockdown of *TbPI-PLC-like expression* by RNAi does not affect IP_6 production in PCF. **a**. HPLC separation profile of IP_6 from *T. brucei*. Inset shows the chemical structure of IP_6. HPLC chromatogram is representative of multiple runs. **b**. Negative ion electrospray mass spectrometry of IP_6 detected in wild type PCF. Mass to charge corresponding ion for IP_6 $[M-2]^{-2}$ (329 *m*/*z*) was detected. **c**. Bar graph scaled in arbitrary units and normalized with a standard, 3-fluoro-IP_3, showing the relative abundance of IP_6 measured using mass spectrometry. Values are means ± s.d. from three different experiments (n = 3); ns, not significant. $P = 0.765$. Student's *t* test.

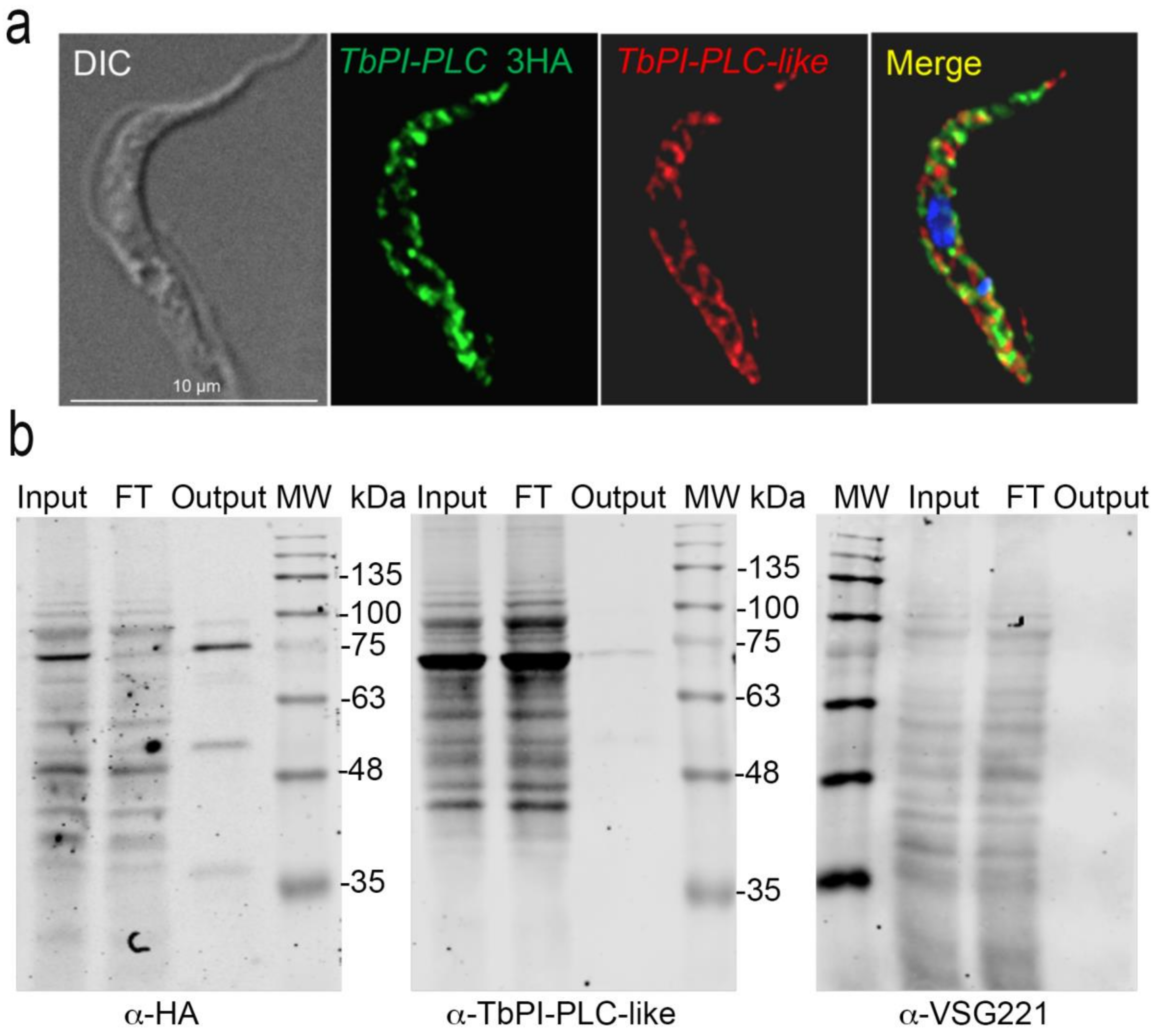

Figure S5. Interaction between TbPI-PLC-HA and TbPI-PLC-like. **a.** HA tagged TbPI-PLC1 (*green*) and TbPI-PLC-like (*red*) have a similar subcellular localization in the cytosol and the plasma membrane of PCF. The two proteins partially colocalize (*Merge*). Pearson correlation coefficient = 0.6574. DIC, differential interference contrast microscopy. Bar = 10 μm. **b**. Western blot analysis of TbPI-PLC1 pulldown with anti-HA agarose beads, using commercial anti-HA antibody, polyclonal TbPI-PLC-like antibody, and TbVSG-221antibody (negative control).

Table S1. Primers used in this study.

***TbPI-PLC-like* heterologous expression in pQE-80L vector**	
Forward (*BamHI*)	CGGGATCCGGTTTGTGCAATTCTAAAAG
Reverse (*HindIII*)	CCCAAGCTTTTAGGCCGCCTTGTTCCAGGAAAC
***TbPI-PLC-like* fragment expressed in p2T7^{Ti}B/GFP vector**	
Forward	GGATCCCTCAACGGAAGAAGGTGCTC
Reverse	CCGCGGCCTCCCTCTCTTGAATTCCCG
**C-terminal tagging in pMOTag vectors**	
TbPI-PLC-like forward	GATACCGCGTTCTGCGCTTGGAGCAGAC-CGGTGGCCTTACAAGGTTCGACGAGTGCGCACTTGGT GCTCACTGCCTAGTTTCCTGGAACAAGGCGGCC GGTACCGGGCCCCCCCTCGAG
TbPI-PLC-like reverse	TACGCTTACACTCCCGATTGGCAACGCCCGTCGTCAC-CTAAAACGAGCGGCCTACGTACATTGCGA ATTTGTTAG-CATACGTAACGCCATCTCACCCCA CTTGGCGGCCGCTCTAGAACTAGTGGAT
TbPI-PLC forward	TTAC-GTGCCCTAAAAAAAGGAATTCGCCAGGTCCCCCTTCGA-GACCTCAAAGGATCTATTATACAT GGCTCTTTTTTAATGGTTCAAGTATCTTATCAGG GTACCGGGCCCCCCCTCGAG
TbPI-PLC reverse	AGCTACGCTGTGAAGACCATGGCACAACATGGTGCCA-TAAAGTCGGGTACATGCAACGCACACTTG TAACCCAATT-GAAGAACACGATATTCTCAAACTC TGGCGGCCGCTCTAGAACTAGTGGAT
**qRT-PCR**	
TbActin forward	GTATAGCGTGTGGATTGGCGG
TbActin reverse	TGCTGTGTACGATGCTGGG
TbPI-PLC forward	TGTACGTCACGTGGGCTCTC
TbPI-PLC reverse	GTGCCTCAACTGCCCCTGTC
TbPI-PLC-like forward	TTTGAGAGAGATCAACTAGC
TbPI-PLC-like reverse	CTCGTAAGCCGCCGGGTTCG
**Yeast Two Hybrid**	
TbPI-PLC-like pGADT7 for-ward	TATGGCCATGGCCAGTGGGGGAAACCGCTTAA GTG
TbPI-PLC-like pGADT7 re-verse	CAGCTCGAGCTCGATGGATCTTAGGCCGCCTTGTTCCAG
TbPI-PLC-like pGBKT7 for-ward	GCATATGGCCATGGAGGCCGGGGGAAACCGCT TAAGTG
TbPI-PLC-like pGBKT7 re-verse	CCGCTGCAGGTCGACGGATCTTAGGCCGCCTT GTTCCAG
TbPI-PLC pGADT7 forward	GCATATGGCCATGGAGGCCGGGCACCGTACCG GCAACC
TbPI-PLC pGADT7 reverse	CCGCTGCAGGTCGACGGATCCTACTGATAAGATACTT-GAACCATTAAAAAAGAGCCATGTATAATAG
TbPI-PLC pGBKT7 forward	TATGGCCATGGAGGCCAGTGGGCACCGTACCG GCAACC
TbPI-PLC pGBKT7 reverse	CAGCTCGAGCTCGATGGATCCTACTGATAAGATACTT-GAACCATTAAAAAAGAGCCATGTATAATAG
